# A Mini-Review on Chitosan-Based Hydrogels with Potential for Sustainable Agricultural Applications

**DOI:** 10.3390/polym12102425

**Published:** 2020-10-21

**Authors:** Regina Michalik, Ilona Wandzik

**Affiliations:** 1 Joint Doctoral School, Silesian University of Technology, Akademicka 2a, 44 100 Gliwice, Poland; regina.michalik@polsl.pl or; 2 Research and Innovation Department, Grupa Azoty Zakłady Azotowe Kędzierzyn S.A., Mostowa 30A, 47 220 Kędzierzyn-Koźle, Poland; 3 Department of Organic Chemistry, Bioorganic Chemistry and Biotechnology, Faculty of Chemistry, Silesian University of Technology, Krzywoustego 4, 44 100 Gliwice, Poland; 4 Biotechnology Center, Silesian University of Technology, Krzywoustego 8, 44 100 Gliwice, Poland

**Keywords:** chitosan, hydrogel, superabsorbent polymer, controlled fertilizer release, water absorbency

## Abstract

Agriculture is an important sector of the economy, but this industry consumes significant amounts of water, which is a precious and limited natural resource. Irrigation techniques and efforts to mitigate water usage influence the growth, survival, and yield of crops. However, superabsorbent polymers in combination with fertilizers can be employed to obtain sustained release of nutrients and improved water retention capacity of the soil. Despite significant recent progress in this area involving synthetic polyacrylate hydrogels, there are no industrially applicable solutions exhibiting similar performance using natural biopolymers or synthetic polymers enriched with natural components. This review focuses on biodegradable chitosan-based hydrogels (both natural and semi-synthetic), and discusses their potential agricultural and horticultural applications. The methods for synthesizing hydrogels via physical or chemical crosslinking, and the resulting functional properties of recently reported hydrogels, such as water retention and release of active ingredients, are presented herein.

## 1. Introduction

Hydrogels are crosslinked polymers with hydrophilic groups, which can absorb a large amount of water without being dissolved in it [1,2,3]. Hydrophilic functional groups attached to the polymer backbone allow water absorption, and the crosslinks between network chains induce resistance to dissolution. When swollen, hydrogels maintain the stability of their networks because of their crosslinked structure, thus allowing them to remain stable in a variety of environments. Some hydrogels can absorb aqueous fluids up to thousands of times their own weight and therefore they are called superabsorbents [4,5]. In general superabsorbent polymer hydrogels are based on ionic monomers and are lightly crosslinked; therefore, they display an exceptional capacity of water absorption, significantly higher than highly crosslinked hydrogels made of nonionic monomers. An ideal hydrogel/superabsorbent for use in the agriculture industry must meet a number of requirements, including high water absorption capacity, adjustable absorption rate, high absorbency when exposed to water, high gel fraction after crosslinking, low cost, excellent stability after swelling and during storage, non-toxicity, and rewettability.

Hydrogel materials are considered soil conditioners and yield enhancers, which are capable of retaining both water and nutrients, and then releasing them over an extended period of time. The rate of nutrient release during hydrogel degradation can be adapted to the nutritional requirements of the plants. Hydrogels are applied as coatings for environmentally-friendly fertilizers and such coated fertilizers can improve the soil’s water retention capacity [6]. Slow-release fertilizer hydrogels are another type of agricultural material, which combine the abilities of a superabsorbent hydrogel and a fertilizer; they improve soil quality and increase fertilizer efficiency. The combination of superabsorbent hydrogels and fertilizers is a growing trend for regulating water and nutrients in one system [7].

Although most superabsorbents are comprised of synthetic polymers (typically acrylic monomers) because of their excellent price/performance ratio, the idea of partially or completely replacing such synthetic materials with “greener” alternatives must be considered for environmental reasons. Biopolymers, such as polysaccharides are an environmentally-friendly alternative to synthetic polymers, because they are cheap, readily available, and renewable organic materials [8,9]. Additionally, their natural origin makes them inherently biocompatible, biodegradable, and non-toxic, so they are often used in the synthesis of hydrogel materials with potential applications in agriculture and horticulture [10,11]. Polysaccharides are widely used in the preparation of hydrogels due to the presence of hydrophilic functional groups, which can absorb water and are easily modified by grafting copolymerization reactions and/or crosslinking using established chemical and physical methods [5,8,10]. Chitosan (CS) [12,13,14], cellulose (Cell) [15,16], starch (ST) [17,18], and alginates (Alg) [19] are among the most important polysaccharides incorporated into hydrogels with potential for large-scale agricultural applications. CS is the most preferred polysaccharide-based raw material for such applications, because apart from being biocompatible and biodegradable, it has antimicrobial properties and can be commercially produced from naturally regenerating resources (e.g., waste seafood shells). General comments on chitosan as a biopolymer and its importance for agriculture are presented in Section 2

Although several methods for synthesizing and employing CS-based hydrogels for various biomedical applications have been developed [20,21], there are limited formulations that meet agricultural requirements. In this review concerning CS-based hydrogels for agricultural use, the main focus is placed on the water absorption properties and the ability to trap and release active ingredients from the hydrogel network. Examples of CS-based hydrogels and their performances are presented in Table 1, and discussed in detail in Section 3. The review covers the literature of the last decade on agricultural applications of CS-based hydrogels and complements other reviews on the extensive use of CS hydrogels, not limited to agricultural sector [6,12,13,14].

## 2. CS as a Biopolymer for Agricultural Applications

CS is a polycationic biopolymer that can be obtained by partial deacetylation of chitin, which is the second most abundant natural polysaccharide. CS is a copolymer consisting of two subunits, D-glucosamine and N-acetyl-D-glucosamine, linked together by β-1,4 glycosidic bonds. It contains two types of reactive functional groups; specifically, a free amino group on the C2 carbon atoms of deacetylated units, and hydroxyl groups on the C3 and C6 carbon atoms on both the acetylated and deacetylated units. These groups facilitate structural modifications of CS, and the generation of functional derivatives that are useful for a variety of applications [22]. The main physicochemical properties that determine CS functionality are its degree of deacetylation (DDA) and its molecular weight. The DDA of commercially available CS ranges from 66% to 95%, and the solubility of CS depends mainly on the DDA value and pH, which determine the ionization state of free amino groups. CS is easily dissolved to make dilute solutions in most organic acids (e.g., acetic acid, formic acid, citric acid) as a result of the protonation of the amino group [23].

CS exhibits antiviral, antifungal, and antibacterial properties in plants, and induces tolerance to abiotic and biotic stress in various horticultural crops [24,25]. The antimicrobial activity of CS varies depending on the degree of deacetylation, molecular weight, and concentration, pH of the solution, viscosity and the target microorganism [26,27].

In addition to demonstrating antimicrobial activity, CS has been employed in soil as a plant nutrient, and has shown high efficacy in combination with other industrial fertilizers without affecting the soil’s natural beneficial microbes [25].

The agricultural use of CS formulations could be diverse ranging from plant protection against various microorganisms, stimulation of plant growth, controlled agrochemical release to pest control and postharvest applications. CS products can be used in various ways, e.g., seed coating agents, soil amendment, foliar spraying agents or supplement in hydroponic [25,26,28]. Due to the multifunctional role of CS in plants, it is increasingly considered as a sensible component of polymer matrices for sustainable agriculture applications. CS is gaining attention as one of the most suitable carriers for agrochemicals. CS alone and its derivatives are capable of forming films, hydrogels, fibres and micro/nanoparticles. Composites of CS with other products, such as gum, starch, and alginate, is a convenient method to improve its properties for slow release of active ingredients.

CS nanoformulations for agrochemicals delivery has received much attention recently [29,30,31,32]. CS-based agronanochemicals represent a sustainable alternative to conventional agrochemicals in crop disease management. The active ingredients can be loaded or encapsulated into CS nanoparticles to form a potent biocide, wherein the resulting non-toxic and biocompatible CS nanoparticles act as a protective wall, shielding plants from the toxic effects of the agrochemicals they contain.

## 3. Strategies for Fabricating CS Hydrogels and Their Potential for Agricultural Applications

Hydrogels are comprised of polymeric chains that are interconnected by crosslinkers, which together form a three-dimensional network. There are several reports on CS-based hydrogels where this network can be generated as a result of chemical (covalent) crosslinking, complexation with an ionic polymer, or by aggregation after CS grafting. In this review CS-based hydrogels are described in the following sections according to the crosslinking method: chemical (Section 3.1) or physical (Section 3.2). Together with the synthetic approach the water absorption properties and other characteristics important for agricultural application are discussed and summarized in Table 1.

### 3.1. CS-Based Hydrogels Fabricated by Chemical (Covalent) Crosslinking

It is relatively easier to control the properties of CS hydrogels produced by covalent crosslinking because their formation and application are not limited by pH. However, their fabrication is more laborious, and contamination with toxic reagents is potentially problematic.

Firstly, hydrogels with superabsorbency characteristics, which could be achieved by incorporation of highly hydrophilic acrylic-based moieties will be presented (Table 1, entry 1–5).

**Figure 1:**
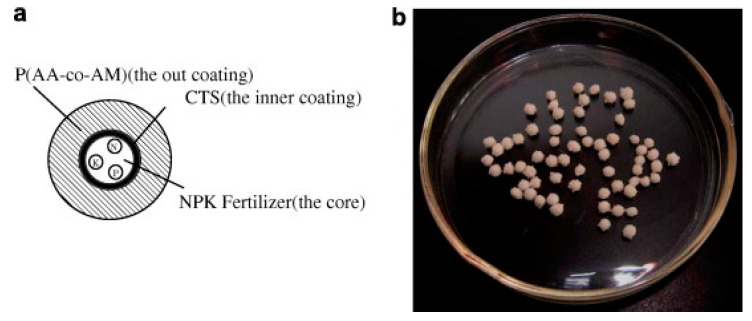
The cross-section schematic view (**a**) and photograph (**b**) of controlled-release and water-retention fertilizer granule. Reprinted with permission from ref. [33] Copyright (2008) Elsevier Ltd.

Superabsorbent polymers have been applied extensively in agriculture and horticulture as water managing materials, and they are mainly fully synthetic. Most commercially available, synthetic superabsorbent polymers are based on polyacrylic acid/polyacrylamide, and they are characterized by water absorbency above 400 g/g. Over the past decade, many researchers have focused on super-swelling multi-component hydrogels with both natural and synthetic component. CS-containing hydrogels are often studied in this context. The combination of slow-release fertilizers and superabsorbent polymers is particularly desirable because they may improve water absorbency and nutrient release into the soil. This approach was described by Wu and Liu [33], who prepared a three-layer system in which the core was a granular nitrogen, phosphorus, and potassium (NPK) fertilizer, the inner coating was CS, and the outer coating was a poly(acrylic acid-co-acrylamide) (poly(AA-co-AM)) superabsorbent polymer (Table 1, entry 1, Figure 1). The water absorbency of the material was 70 g/g, and it retained 25% of the water in the soil after 10 days, 16% after 20 days, and 8% after 30 days. The nutrients released into soil did not exceed 75% by the 30th day.

Another example of a three-layer coated NPK fertilizer hydrogel was developed by Noppakundilograt et al. [34] (Table 1, entry 2). Their multi-component hydrogel was synthesized by coating NPK granules first with polyvinyl alcohol (PVA), and then with CS. The CS layer was then crosslinked using glutaric aldehyde (GA), followed by in situ formation of the outer poly(AA-co-AM) layer.

**Figure 2:**
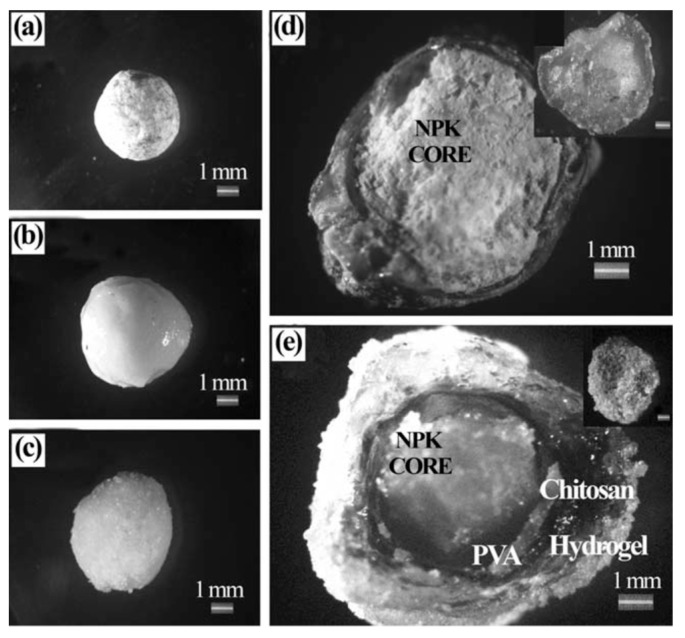
Optical microscope images (3150 magnification) of the various NPK fertilizer granules: (**a**) original (uncoated), (**b**) PVA-coated, (**c**) PVA/CS bilayer-coated, and a cross-section of the (**d**) PVA/cross-CS bilayer coated, and (**e**) PVA/cross-CS/poly(AA-co-AM) three-layer-coated hydrogel from synthesized condition of AA: AM at 97: 3 molar ratios. The inset small figures inserted in (**d**) and (**e**) are the non-cross-section of the two figures themselves. Reprinted with permission from ref. [34] Copyright (2014) Wiley Periodicals, Inc.

Individual granules of the NPK fertilizer at various stages of treatment are shown in Figure 2. Water absorbency of three-layer-coated hydrogel gradually increased with increasing acrylic acid (AA) content. Additionally, swelling of the hydrogel was minimal over the first 15 h, but ultimately increased six-fold after 24 h. The three-layer coated NPK fertilizer hydrogel with the highest water absorption value (233 g/g) demonstrated increased release of N, P, and K nutrients with longer immersion times in the water. The total release after 30 days of immersion in water was 84% (N), 62% (P), and 36% (K). The authors hypothesized that the relatively low percentage of K release may reflect the ionic interaction between the K^+^ ions and the negatively charged carboxylate ions of poly(AA-co-AM) units in the outer layer of the hydrogel.

**Figure 3:**
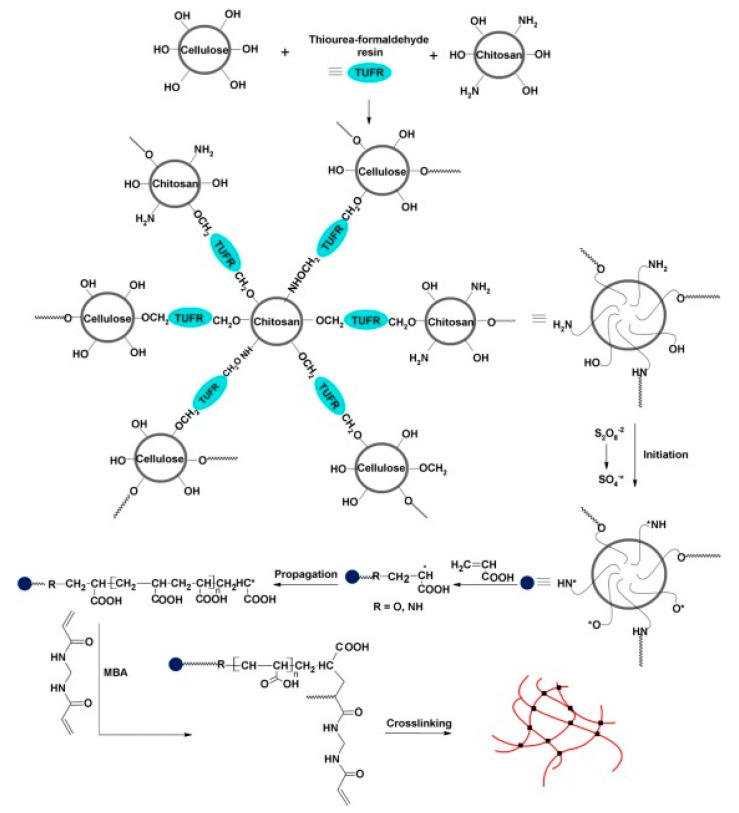
Steps involved in the coupling of cellulose and chitosan into crosslinked backbone (CS/Cell) and successive graft polymerization of acrylic acid from this backbone. Reprinted with permission from ref. [35] Copyright (2016) Elsevier B.V.

Graft copolymerization using chemical or radiation methods is a common approach for preparing CS hydrogels. The structure of a CS graft copolymer has CS as the main chain, and the side chains are polymers composed of grafted monomers, such as AA or AM. Essawy et al. [35] developed superabsorbent polymers fabricated via graft polymerization of AA on a CS-Cell hybrid produced by chemical crosslinking (Table 1, entry 3, Figure 3). The combination of CS and Cell components resulted in a material with enhanced mechanical strength and superior homogenous morphology compared with CS alone. Such CS/Cell hybrids have been prepared via successive polycondensation using thiourea formaldehyde resin. This step was followed by post graft polymerization of AA and formation of the final superabsorbent hydrogel by crosslinking with N’,N-methylenebisacrylamide (MBA). The swelling potential of the superabsorbent composite was evaluated both in water and in a 0.9 wt.% aqueous solution of NaCl. The material demonstrated significantly lower water absorbency in the NaCl solution (39.5 g/g vs. 390 g/g in water) due to the increased ionic strength of the solution, which is a well-known phenomenon. An NPK fertilizer was loaded onto the superabsorbent by immersing the hydrogel in fertilizer solution. The water retention behavior was evaluated under conditions that simulate the soil, and the results showed that 42% and 76% of water in soil had evaporated by the 12th and 24th days, respectively. Additionally, nutrient release from the hydrogel after 3 days was less than 15%, and it did not exceed 75% even after 30 days.

Another example of a CS graft copolymer was presented by Said et al. [36] (Table 1, entry 4). Their superabsorbent composite was fabricated by incorporating ground basaltic rock, as a soil ameliorant, to a hydrogel based on CS grafted onto poly(AA-co-AM). The synthesis was performed via free-radical graft copolymerization under microwave irradiation. Introducing basalt into the hydrogel enhanced its water retention, making it an efficient water-saving material for agricultural applications. The swelling behavior of this composite was pH dependent because of the ionization of amino and carboxylic acid groups; however, over the pH range of 3–8, the water absorbency was at least 500 g/g. Application of the prepared composite as a soil conditioner was investigated using eggplant (*Solanum melongena*) as a model plant. It was observed that the composite hydrogel increased the yield relative to a control experiment without hydrogel.

Radiation-induced crosslinking was introduced as a technique to prepare superabsorbent hydrogels based on polyacrylamide (PAM), CS, and/or Na-Alg [37] (Table 1, entry 5). The water absorbency of such hydrogels depends on the copolymer composition and radiation dose. Specifically, PAM/Alg/CS hydrogels exhibited higher water absorbency relative to PAM/CS, and this behavior was attributed to the ionic character of the Alg component, which enhances swelling. It was observed that the water absorbency decreases with increasing the ionic strength of the solution. At the concentration of 10^−4^ M for Fe^3+^, Ca^2+^ and Na^+^ cation solutions, the water absorbency of PAAm/Alg/CS hydrogel reduces to about 59, 35 and 15% of their water capacity, respectively. Similarly, water absorbency significantly decreases with increasing the concentration of fertilizers, such as ammonium nitrate, ammonium phosphate and ammonium sulfate. The presence of urea causes low reduction in hydrogel water absorbency. It was shown that the presence of studied hydrogels had significant effects on the quality and quantity of maize plants, due to the growth promotion influences of Alg and CS.

As mentioned previously, CS contains two types of reactive functional groups: free amino groups on deacetylated units, and hydroxyl groups on both acetylated and deacetylated units. Depending on the type of cross-linking agent used, the cross-linking reaction involves the participation of an amino or hydroxyl group in the CS chain. Aldehydes are common cross-linking agents that react with the amino groups in CS, forming a covalent imine bond. Several approaches have been developed for synthesizing CS-based hydrogels crosslinked with GA [34,38,39,40,41], glyoxal [42] or salicylic aldehyde [43] for potential agricultural use.

It was determined that blending CS with hydrophilic polymers, such as PVA, can improve the water absorbency of hydrogels. Due to its high hydrophilicity, processability, and biodegradability, PVA is widely used in the agricultural industry to deliver fertilizer and other active ingredients. Controlled release fertilizer hydrogels were prepared from PVA and CS using GA as a crosslinker in the presence of an NPK fertilizer solution. Jamnongkan and Kaewpirom [38] compared the water absorbency and water retention of soil containing CS hydrogels or PVA/CS composites (1:1 by weight), and concluded that both properties were enhanced in the presence of PVA/CS hydrogels (water absorbency = 2.25 g/g; water retention of soil after 30 days = 10%), relative to CS hydrogels (Table 1, entry 6). The authors also evaluated the potassium release behavior of both hydrogels in soil, and determined that CS hydrogels exhibited higher cumulative release of potassium (63% after 30 days). Similar studies regarding PVA/CS hydrogels crosslinked with GA were carried out by Nayan et al. [39], who prepared these materials using a superficial freeze-thawing method (Table 1, entry 7). Optimal mechanical properties and the best swelling ratio of the hydrogels were obtained at 6 wt.% CS loading, and these hydrogels achieved moderate water absorbency (0.58 g/g in phosphate buffered saline (PBS) solution) and water retention in the soil (48% after 15 days). The beneficial effect on the length and weights of the okra plants was observed when hydrogel was incorporated with the soil.

CS hydrogels were also prepared with the aim of encapsulating copper nanoparticles (CuNP), which are believed to be beneficial against certain plant diseases and for inducing antioxidant activity. The use of hydrogels allows greater control over the release of nanoparticles into the soil. CuNP absorbed in CS hydrogels at an optimal concentration of 0.06 g/L had positive effects on tomato growth, yield, and nutritional characteristics [40] (Table 1, entry 8). Similarly, application of CS-PVA hydrogels with absorbed CuNP to grafted watermelon cultivar ‘Jubilee’ plants increased stoma width, primary stem length, and root length [41] (Table 1, entry 9). In both cases, increases in the parameters associated with biomass production was observed even though the concentration of CuNP used was low, suggesting that the presence of chitosan alone has favourable effects.

**Figure 4:**
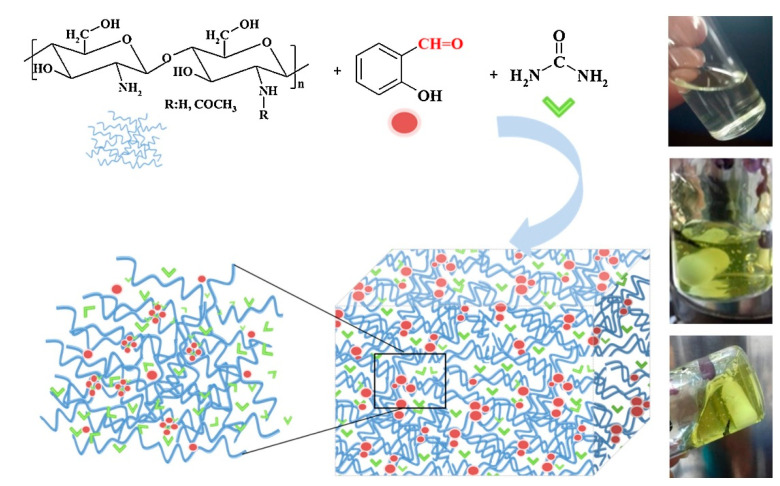
Synthesis of the urea soil conditioners formulations. Reprinted with permission from ref. [43] Copyright (2019) Elsevier Ltd.

New soil conditioner systems were prepared by in situ hydrogelation of CS using salicylaldehyde as a crosslinker in the presence of urea as a fertilizer [43] (Table 1, entry 11, Figure 4). It has been hypothesized that hydrogelation occurs due to ordering of formed imine units into supramolecular clusters, which act as crosslinking nodes. The optimized composition demonstrated high water absorption (68 g/g) and enhanced the water retention capacity of the soil up to 154%. It was evidenced that urea was delivered in three stages governed by the morphological changes of the hydrogel matrix and anchoring forces: firstly, a burst release of 45% in 5 h; then a prolonged release reaching 75% after 11 days and finally release of the residual urea up to the day 35. Preliminary study on tomato seedlings in germination experiments showed that the nitrogen content in the soil increased almost two-fold, and plant growth increased by almost 70% compared to plants cultivated in the reference soil.

In another study CS beads containing nitrification inhibitor—dicyandiamide (DCD) were prepared by precipitation of an acidified chitosan gelling solution and covalently crosslinked with glyoxal [42]. DCD is a nitrification inhibitor that is capable of reducing nitrate (NO^3−^) leaching and nitrous oxide (N_2_O) emissions from soils. It has a relatively short half-life and therefore repeated soil applications are required to maintain efficacy. CS beads encapsulated with DCD were prepared for its controlled released. Slow release of DCD was observed: 19% after 9 h in water; and only 33% after 7 days under the high rainfall conditions.

A major disadvantage of crosslinking CS with aldehydes is their toxicity. Therefore, natural crosslinking agents, which are safer and more environmentally-friendly, have been proposed. Hussain et al. [44] prepared urea-containing CS microspheres via emulsification of chitosan and urea in water-in-oil emulsion followed by crosslinking with genipin (Table 1, entry 12). Several formulations varied by CS, urea and crosslinker concentrations were prepared and the effect of these parameters on urea loading, urea release rate and water uptake was studied. The best water uptake of 1.64 g/g was recorded for microspheres composed of CS:urea weight ratio 0.5:1.0 containing genipin in amount of 0.1 mmol/g of polymer. The corresponding entrapment efficiency was 87% and the urea release into the water was about 90% after 7 days.

Another method for covalent modification of CS is mild oxidation and grafting with itaconic acid (IA), which ultimately results in a combination of chemical, physical, and ionic interactions [45] (Table 1, entry 13). Hydrogels produced in this manner presented high swelling capacities, up to 23 g/g depending on the degree to which they were grafted. The hydrogels were evaluated for controlled-release of urea from the dried films. It was observed that the urea release was very slow at the beginning due to the swelling of the hydrogel, but later, there was an increase in the release rate, up to a maximum of 73% urea released after 50 h.

### 3.2. CS-Based Hydrogels Fabricated by Physical Crosslinking

The reagents used in the chemical crosslinking method are often toxic, so any residual crosslinking agent must be removed before the material can be employed for agricultural applications. Several types of CS-based hydrogels have been prepared by physical crosslinking without using toxic chemical reagents. Due to its free amino groups, CS carries a positive charge and can form hydrogels by reacting with various negatively charged polymers or anionic crosslinking agents. Ionotropic gelation and polyelectrolyte complexation, or a combination of these methods, show great promise for developing controlled-release fertilizers.

In ionotropic gelation, hydrogels are produced based on the ability of CS polycations to crosslink with counterions, forming an ionically crosslinked network. Several CS and CS/ST blends in the form of macrospheres have been prepared for agrochemical applications using sodium tripolyphosphate (TPP) counterions [46,47,48] (Table 1, entries 14–16). Macrospheres were prepared by a simple dripping technique, using an aqueous solution of TPP as the crosslinking agent. Potassium nitrate was entrapped in the hydrogel by immersing the dry hydrogel in a fertilizer solution, and the resulting potassium nitrate-loaded beads were shown to be useful as a controlled-release fertilizer. After 14 days of continuous release into distilled water, the cumulative concentration in the release medium reached between 72% and 95% of the initially-loaded salt concentration [46,48]. It was further shown that these ionically crosslinked CS/ST macrospheres were successfully loaded with plant growth-promoting bacteria, e.g., *Azospirillum brasilense* and *Pseudomonas fluorescens* [47]. It was observed that steam sterilization of the matrix affected size of macrobeads and a significant reduction in the average particle size from 2.97 to 1.82 mm was experienced. Furthermore sterilized macrobeads showed a higher equilibrium degree of swelling (235%) with respect to non-sterilized ones (143%). The authors explained that sterilization process would produce a decrease in the number of inter-macromolecular hydrogen bonding and ionic crosslinking, leading to a significant increase in equilibrium swelling capacity. Produced microbeads were capable of releasing viable cells in a fast and prolonged manner on the non-sterile soil, which has strong potential as biofertilizers based on living microorganisms.

A spray drying technique was used to fabricate microsphere and microcapsule structures of CS and CS/montmorillonite (MMt) clay formulations crosslinked with TPP [49] (Table 1, entry 17). Pure CS microspheres are typically not suitable for controlled fertilization systems due to their high water swelling and immediate release of encapsulated nutrients. However, CS microcapsules, wherein the nutrient was trapped in the core and covered by the additional layer of CS (shell), demonstrated better performances because they first swell, and then release the nutrient. A higher degree of swelling and delayed release of KNO_3_ were observed for microcapsules with a core-shell structure that did not involve crosslinking with TPP.

**Figure 5:**
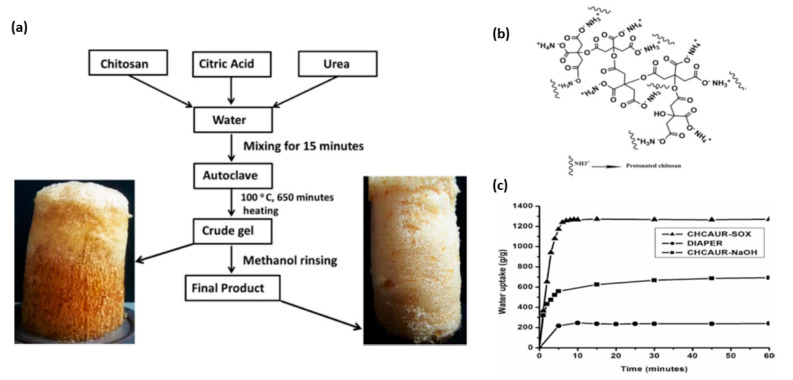
(**a**) Process flow chart representing the preparation of CHCAUR, (**b**) Urea-citric acid adduct oligomer, (**c**) Water absorption versus time for CHCAUR, commercial diaper material and CHCAUR after extraction with sodium hydroxide. Reprinted with permission from ref. [51] Copyright (2018) Elsevier Ltd.

Novel superabsorbent hydrogels were prepared by ionotropic gelation of CS with EDTA-urea and citric acid-urea adducts while heating in aqueous media [50,51] (Table 1, entries 18–19). The authors hypothesized that the water absorption demonstrated by these materials results from the physical crosslinks consisting of electrovalent bonds, macro pores, and other morphological features. The highest water absorbency, which was comparable with other synthetic superabsorbent materials, such as diaper, was obtained for the CS crosslinked with citric acid-urea adduct (CHCAUR, Figure 5) [51]. It was prepared by hydrothermal synthesis at 100 °C in the weight ratio of 1:2:2 (CS: citric acid: urea). The high water absorption of CHCAUR was proposed to result from the porous structure of CHCAUR and the presence of physical crosslinks of urea-citric acid oligomers connecting the CS molecules. Physical crosslinks through ionic bonds could arise due to interaction between protonated CS and postulated urea-citric acid oligomers presented in Figure 5b. CHCAUR was found to absorb 1250 g/g of distilled water and 210 g/g of 0.1% sodium chloride solution. Water absorption versus time for CHCAUR, commercial diaper material and CHCAUR after extraction with sodium hydroxide are presented in Figure 5c. Furthermore it was shown that soil loaded with 4 wt.% of CHCAUR delays the water evaporation from soil, for example after 1 h in 35 °C, the soil with CHCAUR retained 25% of moisture while the control could retain only 10%.

**Figure 6:**
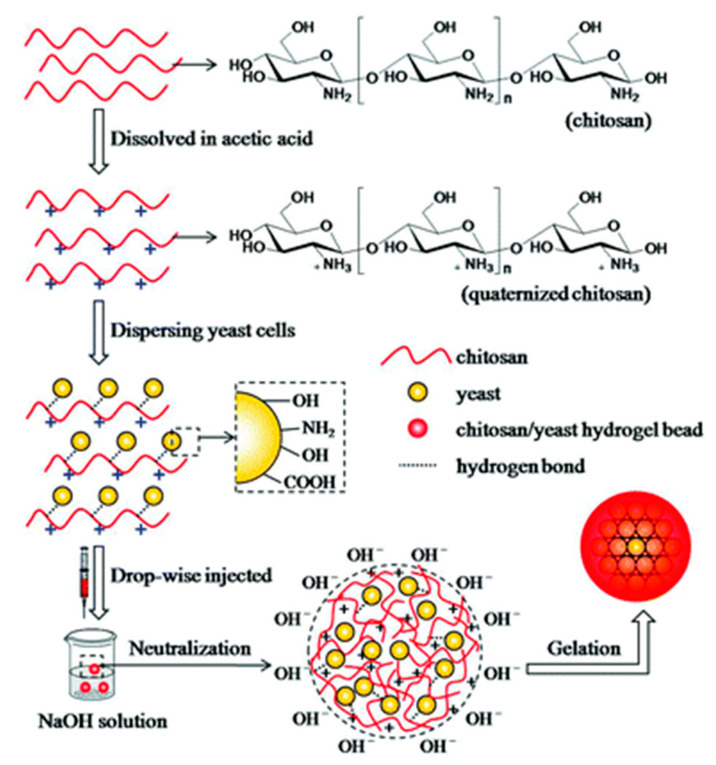
Mechanism of formation of CS/yeast hybrid hydrogel beads. Reprinted with permission from ref. [52] Copyright (2016) The Royal Society of Chemistry and the Centre National de la Recherche Scientifique.

Feng et al. [52] showed that impregnation of yeast cells into the CS matrix could effectively enhance the mechanical stability of CS/yeast hybrid hydrogels (Table 1, entry 20, Figure 6).

The microspheres with 40 wt.% yeast content achieved the maximum swelling ratio of 32 g/g in distilled water. Humic acid was loaded into the hydrogel as a soil supplement, and the slow-release efficiency of humic acid was shown to be pH-dependent, with the optimal 82.6% release value achieved at pH 7 after 5 h.

Recently, López-Velázquez et al. [53] presented ternary blends based on CS, gelatin, and PVA (1:1:1 wt. ratio) for agriculture applications (Table 1, entry 21). Blends were freeze-dried to form junction points in the form of crystallites and interpolymer complexation followed by a physical gelation through alkaline treatment. The hydrogels were characterised by a water absorption from 10.8 to 12 g/g in pH range 5.5–7. Hydrogels loaded with inulin were found to be capable of inducing resistance against *Phytophthora capsica* in chili plants.

Sabadini et al. [54] described the polyelectrolyte complexation of CS with an anionic polymer, gellan gum, which is a heteropolysaccharide produced by fermentation (Table 1, entry 22). The best water absorbency of 219 g/g was obtained for the CS/gellan gum hydrogel with a 1:4 composition. Water evaporation analysis showed that the hydrogel samples retained water for about 9 h, compared to about 4 h for evaporation of pure water. Moreover, the CS/gellan gum samples were tested as commercial monopotassium phosphate (MKP) fertilizer release agents and demonstrated almost complete release in approximately 8 h.

## 4. Conclusions

The goal of sustainable agriculture is to increase productivity, while causing the least possible damage to the environment. Hydrogel technology has allowed development of crosslinked acrylic-based (co)polymers, known as superabsorbent polymers, for agricultural use, and such materials have been used since the late 1980s to improve the physical properties of soil. Currently, the growing concern regarding environmental protection has increased scientific interest in hydrogels of natural origin, or at least enrichment of synthetic materials with natural, biodegradable components. The combination of superabsorbent hydrogels and fertilizers in one system is especially valuable for improving soil quality and increasing fertilizer efficiency. CS hydrogels represent potential materials for this purpose due to their biocompatibility, biodegradability, and non-toxicity. Additionally, CS has antimicrobial properties and could be commercially produced from naturally regenerating resources, such as waste seafood shells.

However, there are some difficulties associated with applying CS hydrogels as commercial products in agriculture. Formulations without incorporation of monomers based on acrylates or acrylamides exhibited moderate water absorbency, not exceeding 70 g/g. The exceptions are hydrogels fabricated via ionotropic gelation of CS by urea adducts of citric acid or EDTA, which demonstrated water absorbency at a level similar to that of synthetic acrylate superabsorbents. The low aqueous solubility, as well as the wide range of deacetylation degree and molecular weight of raw materials may cause synthetic problems during chemical modifications. Water retention capacity and release rate of active ingredients from CS hydrogels are significantly affected by changes in pH, because of the protonation/deprotonation of CS, which is a cationic polyelectrolyte. Moreover, the presence of inorganic fertilizers greatly decreases the water absorbency of such hydrogels.

Despite the discussed drawbacks, CS hydrogels are very attractive materials for agricultural applications, and they can be utilized to deliver and control the release of active ingredients. Such hydrogels embody desirable properties for agricultural products, and can be produced from waste materials without depleting natural resources.

## Figures and Tables

**Table 1:** CS-based hydrogels in controlled release of active ingredients for agricultural applications (2010–2020).

Entry	CS Source/Properties	CS-Based Matrices	Method	Water Absorbency		Fertilizers/Active Ingredients		Ref.
				[g/g]	Performances	Formulation	Performances	
**Polyacrylic acid/polyacrylamide superabsorbent polymers containing CS**								
1	CS (Jinxing Biochemical Co (Zhejiang, China): 600 kDa, DDA 90%	CS/poly(AA-co-AM) three-layer hydrogel	Crosslinking (MBA)	70 (H_2_O)	Water retention of soil 25% after 10 days, 16% after 20 days, and 8% after 30 days	NPK fertilizer granules as the core of the three-layer structures	The nutrients released in soil did not exceed 75% on the 30th day	[33]
2	CS (Seafresh CS): 100–200 kDa, DDA 95%	PVA/cross-CS/poly(AA-co-AM) three-layer hydrogel	Crosslinking (GA, MBA)	233 (H_2_O)	n.d.	NPK fertilizer entrapped in the hydrogel during crosslinking	Release of 84% N, 63% P, and 36% K in water after 30 days	[34]
3	CS (Euromedex, France): DDA 85%	(CS/Cell)-*g*-PAA hydrogel	Crosslinking (thiourea formaldehyde resin, MBA)	390 (H_2_O) 39.5 (0.9 wt.% NaCl)	Evaporation of water in soil 42% and 76% on the 12th and 24th days, respectively	NPK fertilizer entrapped in the hydrogel by immersing dry hydrogel in fertilizer solution	Release of 75% N, 73% P, and 73% K on the 30th day. Release of nutrients after 3 days was below 15% and did not exceed 75% after 30 days	[35]
4	CS (Sigma-Aldrich): medium MW, DDA 75–85%	CS-g-P(AA-co-AM)/Basalt hydrogel	Crosslinking (MBA)	75 (pH 2) 650 (pH 3) 525 (pH 6) 575 (pH 8) 200 (pH 10)	Hydrogel composite retained available water for the plant up to 2 weeks after irrigation	n.d.	Hydrogel composite increased the yield of eggplant (*Solanum melongena*)	[36]
5	CS (Sigma-Aldrich, Germany): high MW, DDA 85%	PAM/CS hydrogel PAM/Alg/CS hydrogel	Radiation-induced crosslinking	*ca*. 380 (H_2_O) *ca*. 150 (1 mM NaCl) *ca.* 50 (1 M NaCl)	Over 35% water retention after 4 days	n.d.	Growth promotion effect of Alg and CS observed based on quality and quantity of maize plants	[37]
**CS-based hydrogels fabricated by chemical crosslinking**								
6	CS (Sigma-Aldrich): High molecular weight 310-375 kDa	CS hydrogel PVA/CS hydrogel	Crosslinking (GA)	CS: 0.7 (H_2_O) PVA/CS 1:1 (wt:wt): 2.25 (H_2_O)	Water retention of soil 4% after 30 days Water retention of soil 10% after 30 days	KNO_3_ entrapped in the hydrogel during crosslinking	Release of K in soil: CS: burst effect in soil, 35% after 2.5 days; prolonged release, 63% after 30 days PVA/CS: burst effect in soil, 22% after 2.5 days; prolonged release, 46% after 30 days	[38]
7	CS (Merck)	PVA/CS hydrogel	Crosslinking (GA)	0.58 (PBS)	Water retention of soil 48% after 15 days	NPK fertilizer entrapped in the hydrogel during crosslinking	Effect on Okra seed germination: better germination energy and growth of young plants	[39]
8	CS (Marine Chemicals, Kerala, India): 200 kDa	CS-CuNP hydrogel	Crosslinking (GA)	3 (H_2_O)	n.d.	Cu nanoparticles absorbed into CS hydrogels	Cu nanoparticles in CS hydrogels at a concentration of 0.06 g/L had positive effects on tomato growth, yield, and nutritional characteristics	[40]
9	CS (Marine Chemicals, Kerala, India): 200 kDa	CS/PVA-CuNP hydrogel	Crosslinking (GA)	n.d.	n.d.	Cu nanoparticles absorbed into CS-PVA hydrogels	Application of Cs-PVA-CuNP to grafted watermelon cultivar ‘Jubilee’ plants increased stoma width, primary stem length, and root length by 7%, 8%, and 14%, respectively	[41]
10	CS	CS hydrogel	Crosslinking (glyoxal)	n.d.	n.d.	DCD (nitrification inhibitor) encapsulation in CS hydrogel beads during CS gelling	Release of DCD in soil: 33% after 7 days under the high rainfall treatment	[42]
11	CS: 314 kDa, DDA 87%	CS hydrogel	Crosslinking (salicylaldehyde)	68 (H_2_O)	Water holding capacity in soil up to 154%	Urea encapsulation during crosslinking	Burst effect ≅ 45% in first 5h; prolonged release ≅ 75% after 11 days, and release of the residual urea up to 35 day	[43]
12	CS (Sigma-Aldrich, USA): medium MW	CS microspheres	Emulsion crosslinking (genipin)	1.64 (H_2_O)	n.d	Urea encapsulation during crosslinking	Urea release in water: about 90% after 7 days	[44]
13	CS (from seafood waste industry): 140 kDa, DDA 75%	CS-*g*-IA hydrogel	Graft copolymerization	23 (H_2_O) 10 (NaCl, 0.9 *w/v*) <2.5 (pH 4), 3.5 (pH 2)	n.d.	Urea loaded into CS films	Urea release in water 73% after 50 h	[45]
**CS-based hydrogels fabricated by physical crosslinking**								
14	CS: medium MW, DDA 81%	CS and CS/ST hydrogel macrospheres	Ionotropic gelation (TPP)	1.21–1.63 (H_2_O)	n.d.	KNO_3_ entrapped in the hydrogel by immersing dry hydrogel in fertilizer solution	KNO_3_ release in water: 73% after 14 days	[46]
15	CS (Sigma-Aldrich, USA): medium MW, DDA 81%	CS/ST hydrogel macrospheres	Ionotropic gelation (TPP)	2.35 (sterilized H_2_O) 1.43 (H_2_O)	n.d.	Plant growth-promoting bacteria were loaded into macrospheres	Bacteria release in soil: bacterial concentration increased progressively during the first 20 days, and then began to decrease after 24 days	[47]
16	CS (Sigma-Aldrich, USA): 190–310 kDa, DDA 81%	CS and CS/ST hydrogel macrospheres	Ionotropic gelation (TPP)	1.21–1.63 (H_2_O)	n.d.	KNO_3_ entrapped in the hydrogel by immersing xerogel beads in fertilizer solution	KNO_3_ release in water: 72–95% after 16 days	[48]
17	CS (Polymar S/A): 180 kDa, DDA 85%	CS/MMt hydrogel microcapsules	Ionotropic gelation (TPP)	1.2 (H_2_O)	n.d.	KNO_3_ encapsulated in the core of microcapsules	Complete KNO_3_ release in water after 2 h	[49]
18	CS: DDA 80%	CS hydrogel	Ionotropic gelation (EDTA–urea adduct)	570 (H_2_O) 100 (0.1% NaCl) 40 (1% NaCl)	n.d.	Urea as an adduct with EDTA	Potential matrix for urea release	[50]
19	CS: 48.7 kDa, DDA 80%	CS hydrogel	Ionotropic gelation (citric acid–urea adduct)	1250 (H_2_O) 210 (0.1% NaCl)	Water retention of soil 25% after 1 h at 35 °C, while the control retained only 10%	Urea as an adduct with citric acid	n.d.	[51]
20	CS (Sinopharm Chemical Reagent Co., Ltd.)	CS/yeast hydrogel microspheres	Alkali gelation	26–32 (H_2_O)	n.d.	Humic acid as a model fertilizer loaded into dry hydrogel microspheres	Slow-release efficiency of humic acid: 82.6% at pH 7 after 5 h.	[52]
21	CS: low MW, DDA 92%	Gelatin/CS/PVA hydrogel	Alkali gelation	11 (H_2_O)	Water retention of soil ≅ 70% after 1 day	Inulin solution was injected into dried hydrogel	The hydrogels loaded with inulin were capable of inducing resistance against *Phytophthora capsica* in chili plants	[53]
22	CS (Aldrich): 480 kDa, DDA 85%	CS/high acetyl gellan gum (HAGG) hydrogel	Polyelectrolyte complexation	71–219 (H_2_O) depending on composition of CS:HAGG (4:1 to 1:4)	Time for complete water loss in gels was 9 h, compared to 4 h for evaporation of pure water	MKP fertilizer entrapped in the hydrogel by immersing dry hydrogel in fertilizer solution	Complete MKP release into the water after 8 h	[54]
23	CS (Polymar Co., Brazil): DDA 74%	CS/humic spheres	Ionotropic gelation (TPP)	n.d.	n.d	Urea encapsulation during gelation	Release of urea in soil: 70% after 7 days	[55]
24	CS (Northern Chemicals and Glasswares Company, Thailand)	Silk Fibroin/Gelatin/CS hydrogel	Self-assembly by solvent casting	3.0–4.2 (H_2_O) depending on CS content	Degree of swelling decreases with increasing CS content	Urea embedded in hydrogels	Urea release in water: about 80% after 13 days	[56]

## References

[1] Mahinroosta M., Jomeh Farsangi Z., Allahverdi A., Shakoori Z. (2018). Hydrogels as intelligent materials: A brief review of synthesis, properties and applications. Mater. Today Chem..

[2] Ahmed E.M. (2015). Hydrogel: Preparation, characterization, and applications: A review. J. Adv. Res..

[3] Ullah F., Othman M.B.H., Javed F., Ahmad Z., Akil H.M. (2015). Classification, processing and application od hydrogels: A review. Mater. Sci. Eng. C.

[4] Zohuriaan-Mehr M.J., Omidian H., Doroudiani S., Kabiri K. (2010). Advances in nonhygienic applications of superabsorbent hydrogel materials. J. Mater. Sci..

[5] Mignon A., De Biele N., Dubruel P., Van Vlierberghe S. (2019). Superabsorbent polymers: A review on the characteristics and application of synthetic, polysaccharide-based, semi-synthetic and “smart” derivatives. Eur. Polym. J..

[6] Chen J., Lu S., Zhang Z., Zhao X., Li X., Ning P., Liu M. (2018). Environmentally friendly fertilizers: A review of materials used and their effects on the environment. Sci. Total Environ..

[7] Ramli R.A. (2019). Slow release fertilizer hydrogels: A review. Polym. Chem..

[8] Guilherme M.R., Aouada F.A., Fajardo A.R., Martins A.F., Paulino A.T., Davi M.F.T., Rubira A.F., Muniz E.C. (2015). Superabsorbent hydrogels based on polysaccharides for application in agriculture as soil conditioner and nutrient carrier: A review. Eur. Polym. J..

[9] Tomadoni B., Casalongue C., Alvarez V.A. (2019). Biopolymer-based hydrogels for agriculture applications: Swelling behavior and slow release for agrochemicals. Polymers for Agri-Food Applications.

[10] Campos E.V.R., de Oliveira J.L., Fraceto L.F., Singh B. (2014). Polysaccharides as safer release systems for agrochemicals. Agron. Sustain. Dev..

[11] Pasqui D., De Cagna M., Barbucci R. (2012). Polysaccharide-based hydrogels: The key role of water in affecting mechanical properties. Polymers.

[12] Cheng B., Pei B., Wang Z., Hu Q. (2017). Advances in chitosan-based superabsorbent hydrogels. RSC Adv..

[13] Pereira A.G.B., Martins A.F., Paulino A.T., Fajardo A.R., Guilherme M.R., Faria M.G.I., Linde G.A., Rubira A.F., Muniz E.C. (2017). Recent advances in designing hydrogels from chitin and chitin and chitin-derivatives and their impact on environment and agriculture: A review. Rev. Virtual de Quim..

[14] Nangia S., Warkar S., Katyal D. (2018). A review on environmental applications of chitosan biopolymeric hydrogel based composites. J. Macromol. Sci. A.

[15] Demitri C., Scalera F., Mdaghiele M., Sannino A., Maffezzoli A. (2013). Potential of cellulose-based superabsorbent hydrogels as water reservoir in agriculture. Int. J. Polym. Sci..

[16] Ma J., Li X., Bao Y. (2015). Advances in cellulose-based superabsorbent hydrogels. RSC Adv..

[17] Ismail H., Irani M., Ahmad Z. (2013). Starch-based hydrogels: Present status and applications. Int. J. Polym. Mater..

[18] Jyothi A.N. (2010). Starch graft copolymers: Novel applications in industry, Compos. Interfaces.

[19] Abd El-Rehim H.A. (2006). Characterization and possible agricultural application of polyacrylamide/sodium alginate crosslinked hydrogels prepared by ionizing radiation. J. Appl. Polym. Sci..

[20] Pella M.C.G., Lima-Tenorio M.K., Tenorio-Neto E.T., Guilherme M.R., Muniz E.C., Rubira A.F. (2018). Chitosan-based hydrogels: From preparation to biomedical applications. Carbohydr. Polym..

[21] Peers S., Montembault A., Ladaviere C. (2020). Chitosan hydrogels for sustained drug delivery. J. Control. Release.

[22] Harish Prashanth K.V., Tharanathan R.N. (2007). Chitin/ chitosan: Modifications and their unlimited application potential—An overview. Trends Food Sci. Technol..

[23] Rinaudo M. (2006). Chitin and chitosan: Properties and applications. Prog. Polym. Sci..

[24] Hidangmayum A., Dwivedi P., Katiyar D., Hemantaranjan A. (2019). Application of chitosan on plant responses with special reference to abiotic stress. Physiol. Mol. Biol. Plants.

[25] Sharif R., Mujtaba M., Rahman M.U., Shalmani A., Ahmad H., Anwar T., Tianchan D., Wang X. (2018). The multifunctional role of chitosan in horticultural crops; a review. Molecules.

[26] Morin-Crini N., Lichtfouse E., Torri G., Crini G. (2019). Applications of chitosan in food, pharmaceuticals, medicine, cosmetics, agriculture, textiles, pulp and paper, biotechnology, and environmental chemistry. Environ. Chem. Lett..

[27] Badawy M.E.I., Rabea E.I. (2011). A Biopolymer chitosan and its derivatives as promising antimicrobial agents against plant pathogens and their applications in crop protection. Int. J. Carbohydr. Chem..

[28] Malerba M., Cerana R. (2018). Recent advances of chitosan applications in plants. Polymers.

[29] Maluin F.N., Hussein M.Z. (2020). Chitosan-based agronanochemicals as a sustainable alternative in crop protection. Molecules.

[30] Mujtaba M., Khawar K.M., Camara M.C., Carvalho L.B., Fraceto L.F., Morsi R.E., Elsabee M.Z., Kaya M., Labidi J., Ullah H. (2020). Chitosan-based delivery systems for plants: A brief overview of recent advances and future directions. Int. J. Biol. Macromol..

[31] Kashyap P.L., Xiang X., Heiden P. (2015). Chitosan nanoparticle based delivery systems for sustainable agriculture. Int. J. Biol. Macromol..

[32] Cota-Arriola O., Cortez-Rocha M.O., Burgos-Hernandez A., Ezquerra-Brauer J.M., Plascencia-Jatomea M. (2013). Controlled release matrices and micro/nanoparticles of chitosan with antimicrobial potential: Development of new strategies for microbial control in agriculture. J. Sci. Food Agric..

[33] Wu L., Liu M. (2008). Preparation and properties of chitosan-coated NPK compound fertilizer with controlled-release and water-retention. Carbohydr. Polym..

[34] Noppakundilograt S., Pheatcharat N., Kiatkamjornwong S. (2015). Multilayer-coated NPK Compound fertilizer hydrogel with controlled nutrient release and water absorbency. J. Appl. Polym. Sci..

[35] Essawy H.A., Ghazy M.B.M., El-Hai F.A., Mohamed M.F. (2016). Superabsorbent hydrogels via graft polymerization of acrylic acid from chitosan-cellulose hybrid and their potential in controlled release of soil nutrients. Int. J. Biol. Macromol..

[36] Said M., Atassi Y., Tally M., Khatib H. (2018). Environmentally friendly chitosan-g-poly (acrylic acid-co-acrylamide)/ground basalt superabsorbent composite for agricultural applications. J. Polym. Environ..

[37] Elbarbary A.M., Abd El_Rehim H.A., El-Sawy N.M., Hegazy E.A., Soliman E.A. (2017). Radiation induced crosslinking of polyacrylamide incorporated low molecular weights natural polymers for possible use in the agricultural applications. Carbohydr. Polym..

[38] Jamnongkan T., Kaewpirom S. (2010). Potassium release kinetics and water retention of controlled-release fertilizers based on chitosan hydrogels. J. Polym. Environ..

[39] Nayan N.H., Hamzah M.S., Tahir A., Rajali A.A., Muslih E.F., Mazlan R. (2018). Development of polyvinyl alcohol/chitosan hydrogel loaded with fertilizer compound: Preparation, properties and effect on seed germination. JST.

[40] Juarez-Maldonado A., Ortega-Ortiz H., Perez-Labrada F., Cadenas-Pliego G., Benavides-Mendoza A. (2016). Cu nanoparticles absorbed on chitosan hydrogels positively alter morphological, production, and quality characteristics of tomato. J. Appl. Bot. Food Qual..

[41] Gomez H.G., Godina F.R., Ortiz H.O., Mendoza A.B., Torres V.R., De La Fuente M.C. (2017). Use of chitosan-PVA hydrogels with cooper nanoparticles to improve the growth of grafted watermelon. Molecules.

[42] Minet E.P., O’Carrol C., Rooney D., Gallagher L., Richards K.G. (2013). Slow delivery of a nitrification inhibitor (dicyandiamide) to soil using a biodegradable hydrogel of chitosan. Chemosphere.

[43] Iftime M.M., Ailiesei G.L., Ungureanu E., Marin L. (2019). Designing chitosan based eco-friendly multifunctional soil conditioner systems with urea controlled release and water retention. Carbohydr. Polym..

[44] Hussain M.R., Devi R.R., Maji T.K. (2012). Controlled release of urea from chitosan microspheres prepared by emulsification and cross-linking method. Iran. Polym. J..

[45] León O., Muñoz-Bonilla A., Soto D., Ramirez J., Marquez Y., Fernández-García M.C.M. (2018). Preparation of oxidized and grafted chitosan superabsorbents for urea delivery. J. Polym. Environ..

[46] Perez J.J., Francois N.J. (2016). Chitosan-starch beads prepared by ionotropic gelation as potential matrices for controlled release of fertilizers. Carbohydr. Polym..

[47] Perez J.J., Francois N.J., Maroniche G.A., Borrajo M.P., Pereyra M.A., Creus C.M. (2018). A novel, green, low-cost chitosan-starch hydrogel as potential delivery system for plant growth-promoting bacteria. Carbohydr. Polym..

[48] Perez Bravo J.J., Francois N.J. (2020). Chitosan/starch matrices prepared by ionotropic gelation: Rheological characterization, swelling behavior and potassium nitrate release kinetics. J. Polym. Environ..

[49] Franca D., Medina A., Messa L., Souza C., Faez R. (2018). Chitosan spray-dried microcapsule and microsphere as fertilizer host for swellable-controlled release materials. Carbohydr. Polym..

[50] Narayanan A., Dhamodharan R. (2015). Super water-absorbing new material from chitosan, EDTA and urea. Carbohydr. Polym..

[51] Narayanan A., Kartik R., Sangeetha E., Dhamodharan R. (2018). Super water absorbing polymeric gel from chitosan, citric acid and urea: Synthesis and mechanism of water absorption. Carbohydr. Polym..

[52] Feng D., Bai B., Wang H., Suo Y. (2016). Enhanced mechanical stability and sensitive swelling performance of chitosan/yeast hybrid hydrogel beads. New J. Chem..

[53] Lopez-Velazquez J.C., Rodriguez-Rodriguez R., Espinosa-Andrews H., Qui-Zapata J.A., Garcia-Morales S., Navarro-Lopez D.E., Luna-Barcenas G., Vassallo-Brigneti E.C., Garcia-Carvajal Z.Y. (2019). Gelatin-chitosan-PVA hydrogels and their application in agriculture. J. Chem. Technol. Biotechnol..

[54] Sabadini R.C., Martins V.C.A., Pawlicka A. (2015). Synthesis and characterization of gellan gum: Chitosan biohydrogels for soil humidity control and fertilizer release. Cellulose.

[55] Araujo B.R., Romao L.P.C., Doumer M.E., Mangrich A.S. (2017). Evaluation of the interactions between chitosan and humics in media for the controlled release of nitrogen fertilizer. J. Environ. Manag..

[56] Rattanamanee A., Niamsup H., Srisombat L.-O., Punyodom W., Watanesk R., Watanesk S. (2015). Role of chitosan on some physical properties and the urea controlled release of the silk fibroin/gelatin hydrogel. J. Polym. Environ..

